# Integration of *in vitro* and *in silico* perspectives to explain chemical characterization, biological potential and anticancer effects of *Hypericum salsugineum*: A pharmacologically active source for functional drug formulations

**DOI:** 10.1371/journal.pone.0197815

**Published:** 2018-06-04

**Authors:** Onur Bender, Eulogio J. Llorent-Martínez, Gokhan Zengin, Adriano Mollica, Ramazan Ceylan, Lucia Molina-García, Maria Luisa Fernández-de Córdova, Arzu Atalay

**Affiliations:** 1 Biotechnology Institute, Ankara University, Ankara, Turkey; 2 Department of Physical and Analytical Chemistry, University of Jaén, Campus Las Lagunillas S/N, Jaén, Spain; 3 Deparment of Biology, Science Faculty, Selcuk University, Campus, Konya, Turkey; 4 Department of Pharmacy, University “G. d’Annunzio” of Chieti-Pescara, Chieti-Italy; Institute of Medical Research and Medicinal Plant Studies, CAMEROON

## Abstract

The genus *Hypericum* is one of the most popular genera in both traditional medicine and scientific platform. This study is designed to provide conceptual insights on the biological potential and chemical characterization of *H*. *salsugineum*, which is endemic to Turkey. The qualitative and quantitative phenolic content of the extracts was characterized by HPLC-ESI-MS^n^. Biological efficiency was investigated by enzyme inhibitory assays (cholinesterases, tyrosinase, amylase, and glucosidase) and anti-cancer efficacy tests (anti-proliferative activities with the iCELLigence technology, colony formation and wound healing scratch assays). Phenolic acids (3-*O*-caffeoylquinic, 5-*O*-caffeoylquinic, and 4-*O*-caffeoylquinic acids) were the predominant group in the studied extracts, although several flavonoids were also detected and quantified. The extracts exhibited good inhibitory effects on tyrosinase and glucosidase, while they had weak ability against cholinesterases and amylase. Computational studies were also performed to explain the interactions between the major phenolics and these enzymes. The extracts displayed significant anti-cancer effects on breast carcinoma cell lines. Our findings suggest that *Hypericum salsugineum* could be valued as a potential source of biologically-active compounds for designing novel products.

## Introduction

The plant kingdom usually presents high levels of polyphenols, which are known for their high biological properties such as antioxidant, antimicrobial, and anticancer. Hence, although the health benefits of phenolic compounds have been reported for decades, studies on this area are still a significant concern for many fields of research, including analytical chemistry, biochemistry, biomedicine, etc. Within this framework, uninvestigated wild plant species are also widely studied to find new sources of valuable phytochemicals and, hence, to combat global health problems including cancer, Alzheimer disease, and diabetes mellitus. In many cases, researchers focus on plants commonly used in folk medicine. However, the information of these species is usually extensive, and focusing on less-known species from the same genus is an interesting option, as their composition and bio(chemical) activity may be similar. From this point, new studies could open further avenues in the design of potential phytopharmaceuticals.

The genus *Hypericum* (Hypericaceae) comprehends nearly 500 species which are widely distributed around the world. These species occur as herbs, shrubs and, infrequently trees. Among them, *H*. *perforatum* L. is the best-known species, used in traditional medicine and in the preparation of dietary supplements [1, 2]. Several authors have recently investigated potential medicinal applications of other *Hypericum* species, such as *H*. *androsaemum*, *H*. *connatum*, *H*. *olympicum*, and *H*. *adenotrichum*, among others [3–5], mainly studying their biological activity. Phenolic compounds usually account for many of the bio(chemical) properties of plant extracts, so exhaustive investigation of the phytochemical composition of the plant extracts is mandatory. Different phenolics have been reported in *Hypericum* species, including catechin, quercetin derivatives, phenolic acids, etc. [6, 7], each of these compounds presenting specific chemical properties. In this work, we aimed to study the phytochemical profile and bioactivity of *H*. *salsugineum* N. Robson & Hub.-Mor., an uncommon species in this genus, providing exhaustive information in order to propose an alternative source of bio-(active) phytochemicals.

*Hypericum salsugineum* Rabson & Hub.-Mor. is among the least studied *Hypericum* species. We have found only a small number of scientific reports regarding this species. To our knowledge, only a little information was provided concerning the phenolic composition (chlorogenic acid, kaemperol, myricetin, quercetin, and quercetin glycosides) [8] and antiherpetic activity [9] of this plant. Maltas et al. carried out more experiments and provided additional data regarding the antioxidant and antibacterial activity of *H*. *salsugineum*, among other *Hypericum* species [10]. This research aims to provide a complete study on this species to date, including identification and quantification of the main polyphenols, enzyme inhibitory assays, and evaluation of anti-cancer effects.

## Materials and methods

### Plant material

Aerial parts of *Hypericum salsugineum* were collected from Konya (Cihanbeyli-Gölyazı, 38°28'31.08"N, 33° 7'36.74"E, 950 m) (at the flowering season in 2015 summer) and air dried at room temperature. Taxonomic identification was confirmed by the senior taxonomist Dr. Murad Aydın Sanda, from the Department of Biology, Selcuk University, Turkey. The dried aerial parts were ground to a fine powder (about 0.2 mm) using a laboratory mill. Then, the air-dried aerial parts (10 g) were macerated with 200 mL of methanol at room temperature (25°C ± 1°C) for 24 hours. The extracts were concentrated to dryness under vacuum at 40 °C by using a rotary evaporator and stored at + 4°C in the dark until use.

For the collection of plants, no specific permits were required for the described field studies. For any locations/activities, no specific permissions were required. All locations where the plants were collected were not privately-owned or protected in any way and the field studies did not involve endangered or protected species.

### Chemicals and reagents

All reagents and standards were of analytical reagent (AR) grade unless stated otherwise. We purchased all phenolic standards from Sigma-Aldrich (St. Louis, MO, USA), and prepared individual stock solutions in ethanol (HPLC grade; Sigma). LC–MS grade acetonitrile (CH_3_CN, 99%; LabScan; Dublin, Ireland) and ultrapure water (Milli-Q Waters purification system; Millipore; Milford, MA, USA) were used for the HPLC-MS analyses.

### Chromatographic conditions

For HPLC analysis, 5 mg of dried extract was re-dissolved in 1 ml of methanol, filtered through 0.45 μm PTFE membrane filters, and ten μL of the solution was injected.

The HPLC system was an Agilent Series 1100, composed of a vacuum degasser, an autosampler, a binary pump, and a G1315B diode array detector (Agilent Technologies, Santa Clara, CA, USA). We used a reversed phase Luna Omega Polar C_18_ analytical column of 150 x 3.0 mm and 5 μm particle size (Phenomenex, Torrance, CA, USA) and a Polar C_18_ Security Guard cartridge (Phenomenex) of 4 x 3.0 mm. The best separation was achieved with a mobile phase of water-formic acid (100:0.1, v/v) and CH_3_CN. The following program was used: a) initial mobile phase, 10% CH_3_CN; b) linear increase from 10% to 25% CH_3_CN (0–25 min); c) 25% CH_3_CN (25–30 min); d) linear increase from 25% to 50% CH_3_CN (30–40 min); e) linear increase from 50% to 100% CH_3_CN (40–42 min); f) 100% CH_3_CN (42–47 min). Then, CH_3_CN percentage was returned to the initial mobile phase, with a 7 min stabilization time. The flow rate was 0.4 ml min^-1^.

The HPLC system was connected to an ion trap mass spectrometer (Esquire 6000, Bruker Daltonics, Billerica, MA, USA) equipped with an electrospray interface. The scan range was set at m/z 100–1200 with a speed of 13,000 Da/s. The ESI conditions were: drying gas (N_2_) flow rate and temperature, 10 mL/min and 365 °C; nebulizer gas (N_2_) pressure, 50 psi; capillary voltage, 4500 V; capillary exit voltage, -117.3 V. We used the auto MS^n^ mode for the acquisition of MS^n^ data, with isolation width of 4.0 m/z, and fragmentation amplitude of 0.6 V (MS^n^ up to MS^4^). Esquire control software and Data Analysis were used for data acquisition and data processing, respectively.

### Quantification of polyphenols

Calibration curves (0.5–100 μg/ml in MeOH) were prepared using the corresponding analytical standards when available in our laboratory. In other cases, an appropriate analytical standard was selected: quercetin, apigenin and mirycetin for the corresponding glycosides; 3-*O*-caffeoylquinic acid for caffeoylquinic acids and derivatives; and p-coumaric acid for its derivative. The quantitation was carried out at 320 nm for phenolic acids and 350 nm for flavonoids, using peak area at the analytical signal in the corresponding UV chromatograms. Total individual phenolic content (TIPC) was defined as the sum of the quantified phenolic compounds.

### Enzyme inhibitory effects

Anti-cholinesterases, anti-tyrosinase, anti-amylase, and anti-glucosidase effects were tested for detecting enzyme inhibitory effects. Detailed experimental procedures were given in the below:

For Cholinesterase (ChE) inhibitory activity assay: Sample solution (2 mg/mL; 50 μL) was mixed with DTNB (5,5-dithio-bis(2-nitrobenzoic) acid, Sigma, St. Louis, MO, USA) (125 μL) and AChE (acetylcholines-terase (Electric ell acetylcholinesterase, Type-VI-S, EC 3.1.1.7,Sigma)), or BChE (butyrylcholinesterase (horse serum butyrylcholinesterase, EC 3.1.1.8, Sigma)) solution (25 μL) in Tris–HCl buffer (pH 8.0) in a 96-well microplate and incubated for 15 min at 25 °C. The reaction was then initiated with the addition of acetylthiocholine iodide (ATCI, Sigma) or butyrylthiocholine chloride (BTCl, Sigma) (25 μL). Similarly, a blank was prepared by adding sample solution to all reaction reagents without enzyme (AChE or BChE) solution. The sample and blank absorbances were read at 405 nm after 10 min incubation at 25 °C. The absorbance of the blank was subtracted from that of the sample and the cholinesterase inhibitory activity was expressed as galanthamine equivalents (mgGALAE/g extract) [11].

For Tyrosinase inhibitory activity assay: Sample solution (2 mg/mL; 25 μL) was mixed with tyrosinase solution (40 μL, Sigma) and phosphate buffer (100 μL, pH 6.8) in a 96-well microplate and incubated for 15 min at 25 °C. The reaction was then initiated with the addition of L-DOPA (40 μL, Sigma). Similarly, a blank was prepared by adding sample solution to all reaction reagents without enzyme (tyrosinase) solution. The sample and blank absorbances were read at 492 nm after a 10 min incubation at 25 °C. The absorbance of the blank was subtracted from that of the sample and the tyrosinase inhibitory activity was expressed as kojic acid equivalents (mgKAE/g extract) [12].

For α-amylase inhibitory activity assay: Sample solution (2 mg/mL; 25 μL) was mixed with α-amylase solution (ex-porcine pancreas, EC 3.2.1.1, Sigma) (50 μL) in phosphate buffer (pH 6.9 with 6 mM sodium chloride) in a 96-well microplate and incubated for 10 min at 37 °C. After pre-incubation, the reaction was initiated with the addition of starch solution (50 μL, 0.05%). Similarly, a blank was prepared by adding sample solution to all reaction reagents without enzyme (α-amylase) solution. The reaction mixture was incubated 10 min at 37 °C. The reaction was then stopped with the addition of HCl (25 μL, 1 M). This was followed by addition of the iodine-potassium iodide solution (100 μL). The sample and blank absorbances were read at 630 nm. The absorbance of the blank was subtracted from that of the sample and the α-amylase inhibitory activity was expressed as acarbose equivalents (mmol ACE/g extract) [13].

For α-glucosidase inhibitory activity assay: Sample solution (2 mg/mL; 50 μL) was mixed with glutathione (50 μL), α-glucosidase solution (from Saccharomyces cerevisiae, EC 3.2.1.20, Sigma) (50 μL) in phosphate buffer (pH 6.8) and PNPG (4-N-trophenyl-α-D-glucopyranoside, Sigma) (50 μL) in a 96-well microplate and incubated for 15 min at 37 °C. Similarly, a blank was prepared by adding sample solution to all reaction reagents without enzyme (α-glucosidase) solution. The reaction was then stopped with the addition of sodium carbonate (50 μL, 0.2 M). The sample and blank absorbances were read at 400 nm. The absorbance of the blank was subtracted from that of the sample and the α-glucosidase inhibitory activity was expressed as acarbose equivalents (mmol ACE/g extract) [14].

### Molecular modelling

#### Enzymes preparation

For the computational studies, the following crystal structures have been downloaded from the Protein Data Bank RCSB PDB [15]: AChE (pdb:4X3C) [16] in complex with tacrine-nicotinamide hybrid inhibitor, BChE (pdb:4BDS) [17] in complex with tacrine, α-amylase (pdb:1VAH) [18] in complex with r-nitrophenyl-α-D-maltoside, α-glucosidase (pdb:3AXI) [19] in complex with maltose, and tyrosinase (pdb:2Y9X) [20] in complex with tropolone. The enzymes have been prepared for docking by removing the non-catalytic waters, the inhibitors, and all the other molecules present in the pdb files, as previously reported in our recent publications [11, 21–23]. The proteins were neutralized at pH 7.4 by PROPKA; seleno-cysteines, and seleno-methionines, if present, were converted respectively to cysteines and methionines. All the missing fragments and other errors present in the crystal structures were automatically solved by the Wizard Protein Preparation implemented in Maestro 10.2 suite [24]; the crystallographic ligands were used to generate the docking grid.

#### Ligands preparation

3-*O*-caffeoylquinic acid, 4-*O*-caffeoylquinic acid, 5-*O*-caffeoylquinic acid, myricetin-*O*-glucoside, quercetin-3-*O*-glucoside, quercetin-3-*O*-galactoside, quercetin-3-*O*-rhamnoside, myricetin, and quercetin were selected as representative compounds to carry out molecular docking studies because these are well known bioactive compounds and are well represented in the extracts of *H*. *salsugineum*. The chemical structures have been downloaded from Zinc database [25] or drawn by Chemdraw software, and used for molecular modelling experiments after preparation. The ligands were prepared by the LigPrep tool embedded in Maestro 10.2, neutralized at pH 7.4 by Epik, and minimized by force field OPLS3 [26].

#### Molecular docking

The performance of Glide XP to dock for ligand docking and scoring on the selected enzymes, was judged by its ability to reproduce the docking poses found of the crystallographic inhibitor. Thus, a series of self-docking experiments were performed previously to run the docking experiments, by preparing the native inhibitors by the Ligand Preparation tool as described above, and by docking them to the respective enzymes. Glide has shown to be largely able to perform a reliable docking for all the selected enzymes by producing a pose within maximum 1.5 Angstroms of RMSD calculated by comparing them with the crystallographic pose [27] Dockings of the selected substances have been performed for each selected enzyme employed for the *in vitro* enzymatic inhibition tests in this work. Glide [28] has been employed for the docking calculations by using the eXtra Precision scoring function for all the enzymes; the binding pocket was determined automatically by centering the grid on the crystallographic inhibitor, extended in a box of 20 Angstroms for each side. The best pose for each compound docked to the selected enzymes was the best ranked.

### Cell lines and culture conditions

Human estrogen receptor-positive MCF-7 and triple negative MDA-MB-231 breast cancer cell lines were obtained from American Type Culture Collection (Rockville, Maryland, USA). Cells were maintained in Dulbecco's Modified Eagle's Medium (DMEM) (Lonza, Basel, Switzerland) supplemented with 10% heat-inactivated fetal bovine serum (FBS) (Biowest, Nuaillé, France), 2 mM L-glutamine, 100 U/ml penicillin, and 100 μg/ml streptomycin at 37°C in a 5% CO_2_ humidified incubator. Cells were routinely passaged every two to three days per week (1:3 ratio). When the cells reached 80% confluency, they were first washed with phosphate buffered saline (PBS), and then trypsinized with 0.25% trypsin-EDTA solution. Total cells were counted using trypan blue dye exclusion method on a hemocytometer before seeding on E-plate L8 or 6-well plate.

### Cytotoxicity and anti-proliferative activity assay using the iCELLigence system

Dynamic monitoring of cytotoxicity and anti-proliferative activity were determined by using iCELLigence real-time cell analysis technology (ACEA Biosciences, San Diego, CA, USA) as previously described [29]. Briefly, after a background reading with 200 μl growth medium on iCELLigence E-plate L8, 100 μl of MCF-7 and MDA-MB-231 cells were seeded at a density of 5.0 x 10^4^ per well. The system was then set to take impedance measurements every 30 min for 72 h. After culturing the cells for 24 h, they were treated with *H*. *salsugineum* methanolic extract between the range of 62.5–2000 μg/ml. The extract was dissolved in growth medium containing %0,1 DMSO for each experiment and all doses were prepared by serial dilution from a 2 mg/ml stock concentrate. In all experiments, %0,1 DMSO and %5 DMSO were used as negative control and positive controls, respectively. The data was recorded and analyzed by iCELLigence software integrated within the system. IC_50_ values (μg/ml) were calculated according to the values at the end of 72 h of all treatment doses. Each assay was performed in duplicate and the IC_50_ values shown are the average of at least three independent experiments.

### Colony formation assay

Colony formation assay allows testing long-term effects of experimental therapeutics on cell colonies [30]. MCF-7 and MDA-MB-231 cells were plated into 6-well tissue culture plates (3.0 x 10^3^ per well) and allowed to adhere for 24h. The cells were then treated with 62.5, 125, 250, and 350 μg/ml of *H*. *salsugineum* methanolic extract. %0,1 DMSO was used as control. Following treatment, plates were incubated for 14 days at 37°C in 5% CO_2_ humidified incubator. Next, the cells were gently washed with ice-cold PBS and fixed with MeOH:acetic acid (3:1, v:v) solution for 5 min at room temperature. Following removal of the fixative solution, they were stained with %0,05 crystal violet for 15 min at room temperature. The plates were washed with double-distilled water and left to dry overnight at room temperature. Colonies containing >50 cells were counted under a stereomicroscope Leica MZ16 A (Wetzlar, Germany).

### Wound healing scratch assay

Wound healing assay is performed to test the migration ability of cells by measuring their motility [31]. MCF-7 and MDA-MB-231 cells were seeded in 6-well tissue culture plates at a density of 1.0 x 10^6^ cells/well and cultured in growth medium for 24h. Then, the cells were scratched by a sterile 200 μl pipette tip. Cellular debris was removed and cells were washed with fresh growth medium gently. The cells were incubated with 350 μg/ml *H*. *salsugineum* methanolic extract for 48h. %0.1 DMSO was used as control. Wounded areas were photographed under an inverted microscope Leica DM IL LED (Wetzlar, Germany) at time points 0 and 48 h after scratching. Images were processed and quantified using the ImageJ software (National Institutes of Health, USA).

### Statistical analysis

IC_50_ values were calculated using the iCELLigence software. IC_50_ values, colony formation, and wound closure rates are shown as means ± standard deviations (means ± SD). The statistical analyses were performed using the t-test to compare differences in results between the control and treatment groups. P<0.05 was considered to be statistically significant.

## Results and discussion

### HPLC-ESI-MS^n^

**Fig 1:**
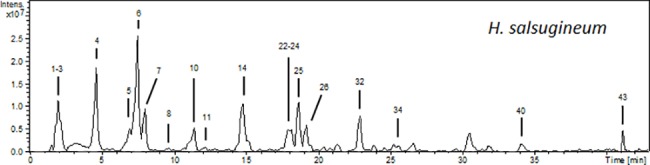
HPLC-ESI/MS^n^ base peak chromatogram (BPC) of the methanolic extract from *H*. *salsugineum*.

We carried out the identification of phenolics (and other minor compounds) in *H*.*salsugineum* by HPLC-ESI-MS^n^ using negative ionization mode. Three independent solutions of the dried extract were analyzed, obtaining similar profiles (see characterization in Table 1). The base peak chromatogram of the methanolic extract of the plant is shown in Fig 1.

**Table 1:** Characterization of methanol extract of *H*. *salsugineum*.

N^o^.	t_*R*_ (min)	[M-H]^-^ *m/z*	m/z (% base peak)	Assigned identification
1	1.8	377	MS^2^ [377]: 341 (100) MS^3^ [377→341]: 179 (100), 161 (70), 131 (7), 119 (72)	Saccharide (derivative)
2	1.8	191	MS^2^ [191]: 173 (20), 127 (12), 111 (100)	Quinic acid *
3	2.0	451	MS^2^ [451]: 449 (48), 353 (100) MS^3^ [451→353]: 191 (100), 179 (19), 173 (16), 135 (12)	Caffeoylquinic acid derivative
4	4.6	353	MS^2^ [353]: 191 (100)	trans-5-*O*-caffeoylquinic acid *
5	7.0	337	MS^2^ [337]: 163 (100) MS^3^ [337→163]: 119 (100)	3-*p*-coumaroylquinic acid
6	7.5	353	MS^2^ [353]: 191 (100)	3-*O*-caffeoylquinic acid *
7	8.0	353	MS^2^ [353]: 191 (26), 179 (61), 173 (100) MS^3^ [353→173]: 111 (100)	4-*O*-caffeoylquinic acid *
8	9.7	431	MS^2^ [431]: 385 (100) MS^3^ [431→385]: 223 (59), 205 (100), 161 (50), 153 (79)	Roseoside (formate adduct)
9	10.0	609	MS^2^ [609]: 463 (69), 447 (88), 301 (100) MS^3^ [609→301]: 271 (100), 179 (48), 151 (99)	Quercetin-*O*-hexoside-*O*-deoxyhexoside
10	11.4	337	MS^2^ [337]: 173 (100), 191 (29) MS^3^ [337→173]: 111 (100)	4-*p*-coumaroylquinic acid
11	12.2	371	MS^2^ [371]: 249 (100), 231 (7) MS^3^ [371→249]: 231 (54), 113 (100), 111 (38)	Unknown
12	12.8	367	MS^2^ [367]: 191 (100), 173 (30) MS^3^ [361→191]: 127 (100)	5-feruloylquinic acid
13	12.9	631	MS^2^ [631]: 479 (100) MS^3^ [631→479]: 317 (100), 316 (54), 179 (9) MS^4^ [631→479→317]: 271 (100), 179 (72), 151 (55)	Myricetin-*O*-(*O*-galloyl)hexoside
14	14.7	479	MS^2^ [479]: 317 (100), 316 (93) MS^3^ [479→317]: 271 (100), 179 (83), 151 (65)	Myricetin-*O*-hexoside
15	15.1	367	MS^2^ [367]: 191 (28), 179 (100), 135 (67) MS^3^ [367→179]: 135 (100)	Methyl-caffeoyl-quinate
16	15.9	479	MS^2^ [479]: 313 (100) MS^3^ [479→313]: 241 (19), 169 (100), 125 (20)	Gallic acid derivative
17	16.3	593	MS^2^ [593]: 503 (95), 485 (19), 473 (37), 413 (25), 383 (100) MS^3^ [593→383]: 355 (100)	Vicenin-2*
18	16.5	521	MS^2^ [521]: 359 (100) MS^3^ [521→359]: 344 (100) MS^4^ [521→359→344]: 329 (26), 313 (28), 300 (49), 189 (78), 159 (100)	Polymethoxylated flavonoid-*O*-hexoside
19	16.6	563	MS^2^ [563]: 443 (8), 413 (100), 293 (85) MS^3^ [563→413]: 293 (100) MS^4^ [563→413→293]: 175 (100)	Apigenin-*C*-hexoside-*O*-pentoside
20	16.9	549	MS^2^ [549]: 503 (100), 371 (45) MS^3^ [549→503]: 371 (100), 161 (13) MS^4^ [549→503→371]: 161 (100), 113 (17)	Unknown
21	17.0	641	MS^2^ [641]: 479 (100), 317 (25) MS^3^ [641→479]: 317 (100) MS^4^ [641→479→317]: 179 (65), 151 (100)	Myricetin-*O*-dihexoside
22	17.7	609	MS^2^ [609]: 301 (100), 300 (20) MS^3^ [609→301]: 271 (100), 255 (54), 179 (51)	Rutin *
23	17.9	463	MS^2^ [463]: 317 (76), 316 (100) MS^3^ [463→316]: 271 (100), 179 (80), 151 (56)	Myricetin-*O*-deoxyhexoside
24	17.9	323	MS^2^ [323]: 323 (100), 242 (51), 241 (24) MS^3^ [323→242]: 240 (100), 172 (39)	Unknown
25	18.6	463	MS^2^ [463]: 301 (100), 300 (20) MS^3^ [463→301]: 179 (69), 151 (100)	Quercetin-*O*-hexoside
26	19.2	463	MS^2^ [463]: 301 (100) MS^3^ [463→301]: 255 (21), 179 (79), 151 (100)	Quercetin-*O*-hexoside
27	19.5	415	MS^2^ [415]: 225 (43), 179 (100), 161 (23), 149 (12) MS^3^ [415→179]: 161 (100), 143 (24), 131 (44), 119 (44)	Saccharide (derivative)
28	20.4	517	MS^2^ [517]: 209 (100)	Unknown
29	20.8	415	MS^2^ [415]: 179 (100) MS^3^ [415→179]: 161 (100), 143 (86), 131 (84), 119 (23)	Saccharide (derivative)
30	21.3	433	MS^2^ [433]: 301 (100), 300 (70) MS^3^ [433→301]: 271 (100), 255 (78), 179 (56), 151 (84)	Quercetin-*O*-pentoside
31	21.3	447	MS^2^ [447]: 285 (100), 284 (96), 255 (21), 151 (7)	Kaempferol-*O*-hexoside
32	22.9	447	MS^2^ [447]: 301 (100), 300 (17) MS^3^ [447→301]: 179 (100), 151 (58)	Quercetin-*O*-deoxyhexoside
33	23.8	445	MS^2^ [445]: 269 (100), MS^3^ [445→269]: 225 (100)	Apigenin glucuronide
34	25.6	317	MS^2^ [317]: 179 (100), 151 (38)	Myricetin
35	26.5	559	MS^2^ [559]: 433 (100)	Unknown
36	27.0	431	MS^2^ [431]: 285 (100) MS^3^ [431→285]: 241 (100)	Luteolin-*O*-deoxyhexoside
37	27.3	625	MS^2^ [625]: 479 (100), 317 (15), 316 (15) MS^3^ [625→479]: 317 (91), 316 (100) MS^4^ [625→479→316]: 271 (100), 179 (24), 151 (52)	Myricetin-*O*-deoxyhexoside-*O*-hexoside
38	29.4	523	MS^2^ [523]: 313 (100) MS^3^ [523→313]: 169 (100) MS^4^ [523→313→169]: 125 (100)	Gallic acid derivative
39	30.4	543	MS^2^ [543]: 417 (100)	Unknown
40	34.0	301	MS^2^ [301]: 179 (71), 151 (100)	Quercetin *
41	35.5	547	MS^2^ [547]: 313 (100) MS^3^ [547→313]: 169 (100), 125 (25) MS^4^ [547→313→169]: 125 (100)	Gallic acid derivative
42	38.6	327	MS^2^ [327]: 291 (32), 229 (100), 211 (44), 171 (75)	Oxo-dihydroxy-octadecenoic acid
43	40.1	329	MS^2^ [329]: 229 (76), 211 (100), 171 (39)	Trihydroxy-octadecenoic acid
44	41.0	537	MS^2^ [537]: 443 (100), 417 (8), 385 (50), 151 (39)	Biflavone
45	42.1	537	MS^2^ [537]: 443 (34), 417 (14), 399 (15), 375 (100) MS^3^ [537→375]: 331 (100)	Amentoflavone

*Identified by comparison with analytical standards

The initial step for the characterization of each compound consisted in the determination of its molecular weight: the base peak corresponded to the deprotonated molecular ion [M-H]^-^, except for one formate adduct. For the identification of the flavonoid glycosides, we compared the mass spectra of the aglycones with analytical standards when available (apigenin, kaempferol, luteolin, and quercetin). Rutin and caffeoylquinic acids were identified by comparison with analytical standards. When reference compounds were not available, we compared the experimental spectra with data from scientific literature.

#### Phenolic acids

Four caffeoylquinic acids were present in the analyzed extracts. Compounds **4**, **6**, and **7** exhibited deprotonated molecular ions at m/z 353. We characterized them by comparison with analytical standards. Compounds **4** and **6**, with the typical 353→191 transition, corresponded to neochlorogenic acid (trans-5-*O*-caffeoylquinic acid) and chlorogenic acid (3-*O*-caffeoylquinic acid), respectively. Compound **7** presented MS^2^[353] base peak at m/z 173, which is characteristic of 4-*O*-caffeoylquinic acid [32]. Compound **3** displayed MS^3^ [451→353] base peak at m/z 191, so we characterized it as a caffeoylquinic acid derivative. Finally, compound **15**, with [M-H]^-^ at m/z 367, was characterized as a methylated derivative according to bibliographic information [33].

We observed two coumaroylquinic acids and identified them according to the hierarchical scheme proposed by Clifford, Johnston (32). Both compounds (**5** and **10**) presented [M-H]^-^ at m/z 337, but different fragmentations. Compound **5** corresponded to 3-*p*-coumaroylquinic acid (MS^2^ base peak at m/z 163), whereas **10** was 4-*p*-coumaroylquinic acid (MS^2^ base peak at m/z 173).

Compound **12** exhibited deprotonated molecular ion at m/z 367 and base peak at m/z 191, a fragmentation pattern that corresponds to 5-feruloylquinic acid [32].

We detected three gallic acid derivatives, compounds **16**, **38**, and **41** –all of them presented the characteristic 169→125 fragmentation of gallic acid–but a complete identification could not be carried out.

#### Flavonoids

A high number of the identified compounds were flavonoids, most of them *O*-glycosides, which we will classify by the aglycone. The identification of the attached moieties (sugars) to the aglycone was based on the observed neutral losses such as hexosyl (162 Da), deoxyhexosyl (146 Da), glucuronyl (176 Da), and pentosyl (132 Da).

Six quercetin glycosides were detected. Compound **40** corresponded to the aglycone (comparison with an analytical standard). Compound **9** suffered neutral losses of 162 Da and 146 Da, so it corresponded to quercetin-*O*-hexoside-*O*-deoxyhexoside. Compounds **25** and **26** were quercetin-*O*-hexoside isomers due to the loss of 162 Da. Similarly, we identified compounds **30** and **32** as quercetin-*O*-pentoside and quercetin-*O*-deoxyhexoside, respectively.

Five myricetin conjugates were characterized, with myricetin aglycone—[M-H]^-^ at m/z 317 and characteristic fragment ions at m/z 179 and 151 –corresponding to compound **34**. Compound **13**, with a deprotonated molecular ion at m/z 631, suffered neutral losses of galloyl (152 Da) and hexosyl moieties 152 Da; it was myricetin-*O*-(*O*-galloyl)hexoside according to bibliography [34]. We characterized compound **14** as myricetin-*O*-hexoside, **21** as myricetin-*O*-dihexoside, **23** as myricetin-*O*-deoxyhexoside, and **37** as myricetin-*O*-deoxyhexoside-*O*-hexoside.

We detected three apigenin glycosides. Compound **17** presented deprotonated molecular ion at m/z 593, and its fragmentation pattern agreed with that of 6,8-di-*C*-glycosyl apigenin (vicenin-2) [35]. Compound **19**, with [M-H]^-^ at m/z 563, presented fragment ions at m/z 413 and 293; we thus characterized it as an apigenin-*C*-hexoside-*O*-pentoside [36]. Finally, compound **33** displayed the neutral loss of 176 Da (glucuronide) to yield the aglycone at m/z 269, so we identified it as apigenin-*O*-glucuronide.

Compound **18** presented [M-H]^-^ at m/z 521, and suffered neutral losses of hexosyl (521→359) and methoxyl (359→344, 344→329) moieties. This fragmentation pattern is consistent with polymethoxylated flavonoid hexosides.

Compound **31** and **36** corresponded to kaempferol-*O*-hexoside and luteolin-*O*-deoxyhexoside, respectively. The aglycones were characterized by comparison with analytical standards.

Finally, two biflavones were characterized. Compounds **43** and **45** presented the deprotonated molecular ions at m/z 537. On the one hand, the fragmentation pattern of compound **45** agreed with amentoflavone [37]. On the other hand, compound **44** was characterized as a biflavone, possibly biapigenin [38].

#### Other compounds

Compounds **1**, **27**, and **29** presented MS^n^ fragment ions at m/z 179, 161, 119, and 131, which have been previously reported in saccharides [39, 40]. We thus characterized these compounds as saccharides or saccharide derivatives.

Compound **8** was a formate adduct and presented the same fragmentation pattern than drovomifoliol-O-β-D-glucopyranoside, a terpenoid described by Li, Zhang [41], and reported as vomifoliol glucoside or roseoside by other authors [34].

We characterized tentatively compounds **42** and **43** as oxo-dihydroxy-octadecenoic and trihydroxy-octadecenoic acids, respectively, considering data from the scientific literature [42, 43].

### Quantification of polyphenols

The concentrations of individual and total phenolics (TIPC) found in the analyzed extracts are shown in Table 2. It can be observed that phenolic acids represented the highest levels of phenolic compounds in *H*. *salsugineum* methanolic extract (approximately 80% of TIPC). The most abundant compounds were 3-*O*-caffeoylquinic acid (73 mg/g DE), 5-*O*-caffeoylquinic acid (51 mg/g DE) and a caffeoylquinic acid derivative (67 mg/g DE). The second most abundant group of compounds corresponded to flavonols, which were mainly composed of myricetin-*O*-hexoside (12.5 mg/g DE) and quercetin-*O*-hexoside isomers (19.2 mg/g DE). Finally, TIPC was 249 mg/g DE.

**Table 2:** Quantification of phenolic compounds in *H*. *salsugineum* (mg g^-1^ DE).

N^o^.	Assigned identification	Concentration (mg g^-1^ DE)
*Phenolic acids*		
3 4	Caffeoylquinic acid derivative 5-*O*-caffeoylquinic acid	67 ± 4 51 ± 2
6	3-*O*-caffeoylquinic acid	73 ± 2
7	4-*O*-caffeoylquinic acid	10.5 ± 0.7
10	4-*p*-coumaroylquinic acid	5.1 ± 0.5
**Total**		**207 ± 5**
*Flavonols*		
14	Myricetin-*O*-hexoside	12.5 ± 0.9
25	Quercetin-*O*-hexoside	15.4 ± 0.8
26	Quercetin-*O*-hexoside	3.8 ± 0.3
32	Quercetin-*O*-deoxyhexoside	6.4 ± 0.3
34	Myricetin	0.83 ± 0.07
40	Quercetin	1.7 ± 0.1
**Total**		**41 ± 1**
*Flavones*		
33	Apigenin	0.3 ± 0.1
43	Biflavone	0.64 ± 0.05
**Total**		**0.9 ± 0.1**
**TIPC**		**249 ± 6**

The concentrations of phenolic acids, as well as TIPC, compare favorably with the levels found in other *Hypericum* species. Jabeur, Tobaldini [44] reported a TIPC value of 110 mg/g in *H*. *androsaemum* extracts, approximately half the levels here observed. For 3-*O*-caffeoylquinic acid (3-CQA) and 5-*O*-caffeoylquinic acid, they observed concentrations of 11.6 and 40.1 mg/g extract, lower than the ones here observed. The levels of quercetin-*O*-hexoside isomers were similar in both cases. However, both the TIPC and phenolic acid concentrations reported in our studies are higher than those found in *H*. *androsaemum*. In *H*. *undulatum* [45], the levels of 3-CQA were also significantly lower (10–16 mg/g) than in the analyzed extracts of *H*. *salsugineum*. In *H*. *perforatum* shoots, only 3.1 mg/g of 3-CQA were reported [46], whereas quercetin hexoside and total flavonoid levels were also lower. Finally, 3-CQA levels in aerial parts of *H*. *origanifolium*, *H*. *montbretii*, and *H*. *perforatum* [47] were lower than the levels found in the present work, except for leaves of *H*. *montbretti*, which were 180 mg/g.

### Enzyme inhibitory effects and molecular modelling

Natural enzyme inhibitors are gaining interest to combat global health problems including Alzheimer’s disease, Diabetes mellitus, hyperpigmentation, and hypertension. The prevalence of these diseases is critically increasing worldwide, and thus effective strategies are required to control these diseases. With this in mind, the discovery of natural and safe enzyme inhibitors is one of the most investigated subjects in the scientific platform. For this purpose, we tested enzyme inhibitory effects of *H*. *salsugineum* extract against cholinesterase, tyrosinase, amylase, and glucosidase. The results are illustrated in Table 3. The tested extract possessed considerable inhibitory potential on tyrosinase and glucosidase. However, the extract exhibited weak ability on cholinesterases and amylase. Although several studies were performed on the enzyme inhibitory properties of some *Hypericum* species [48–50], this work provides the first information concerning *H*. *salsugineum*. The observed activity of the extract might be explained taking into account its phytochemical composition. Apparently, the phytochemical analysis of the extract evidenced the presence of several bioactive compounds. In this context, molecular docking was performed to understand possible interactions between phytochemicals and enzymes. The most abundant substances were selected as targets in the molecular modelling studies.

**Table 3:** Enzyme inhibitory effects of *H*. *salsugineum*.

Assays	Results
Acetylcholinesterase (mgGALAE/g)	1.689±0.150*
Butyrlcholinesterase (mgGALAE/g)	0.244±0.029
Tyrosinase (mgKAE/g)	65.29±0.41
α- Amylase (mmolACAE/g)	0.616±0.073
α-Glucosidase (mmolACAE/g)	19.466±0.704

*Values expressed are means ±S.D. of three parallel measurements. GALAE: Galantamine equivalent; KAE: Kojic acid equivalent; ACAE: Acarbose equivalent.

**Fig 2:**
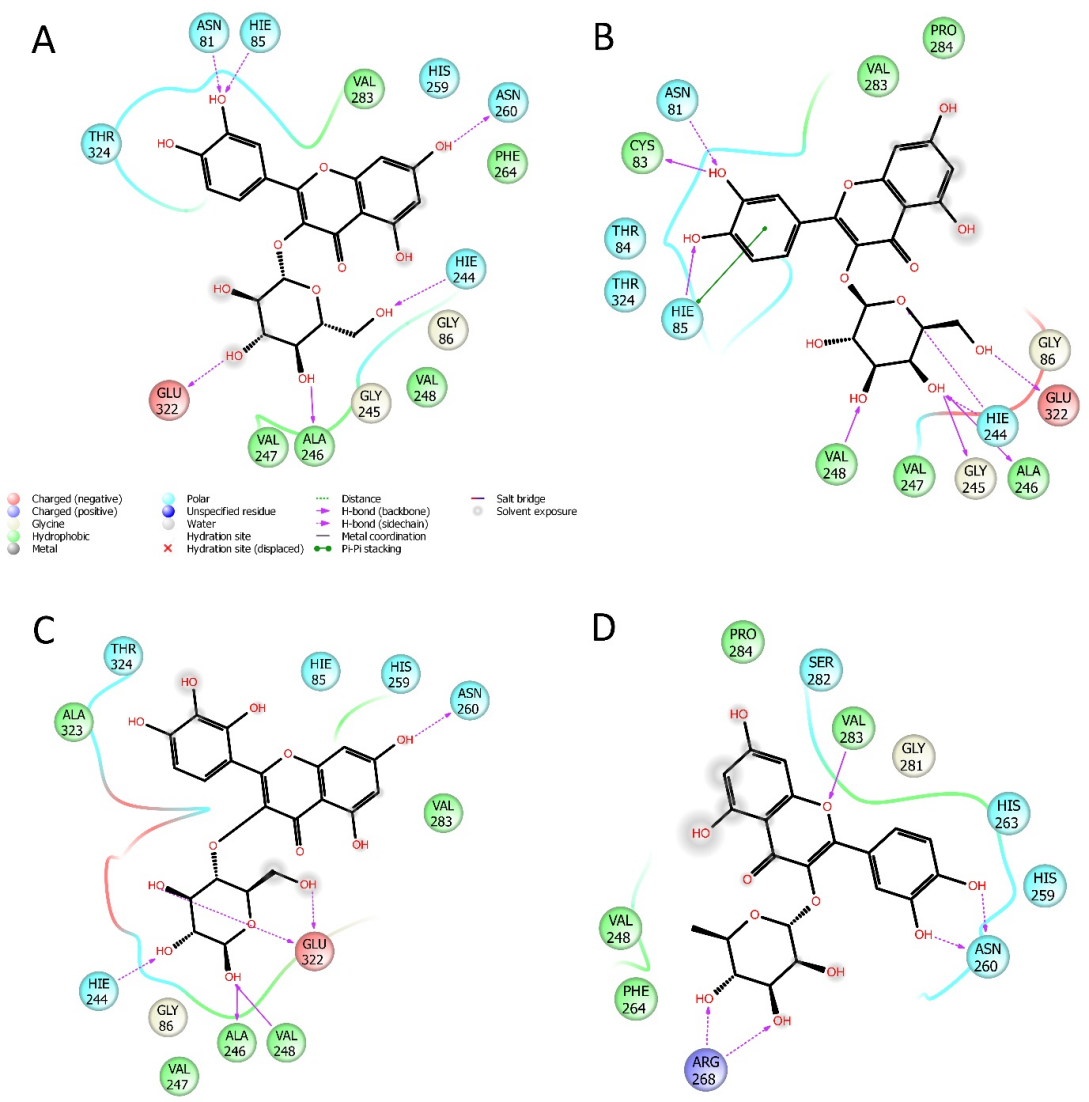
2D representation of the best pose of (A) isoquercitrin, (B) isoquercetin, (C) myricetin-3-O-glucoside, (D) quercitrin docked in the catalitic pocket of tyrosinase.

**Fig 3:**
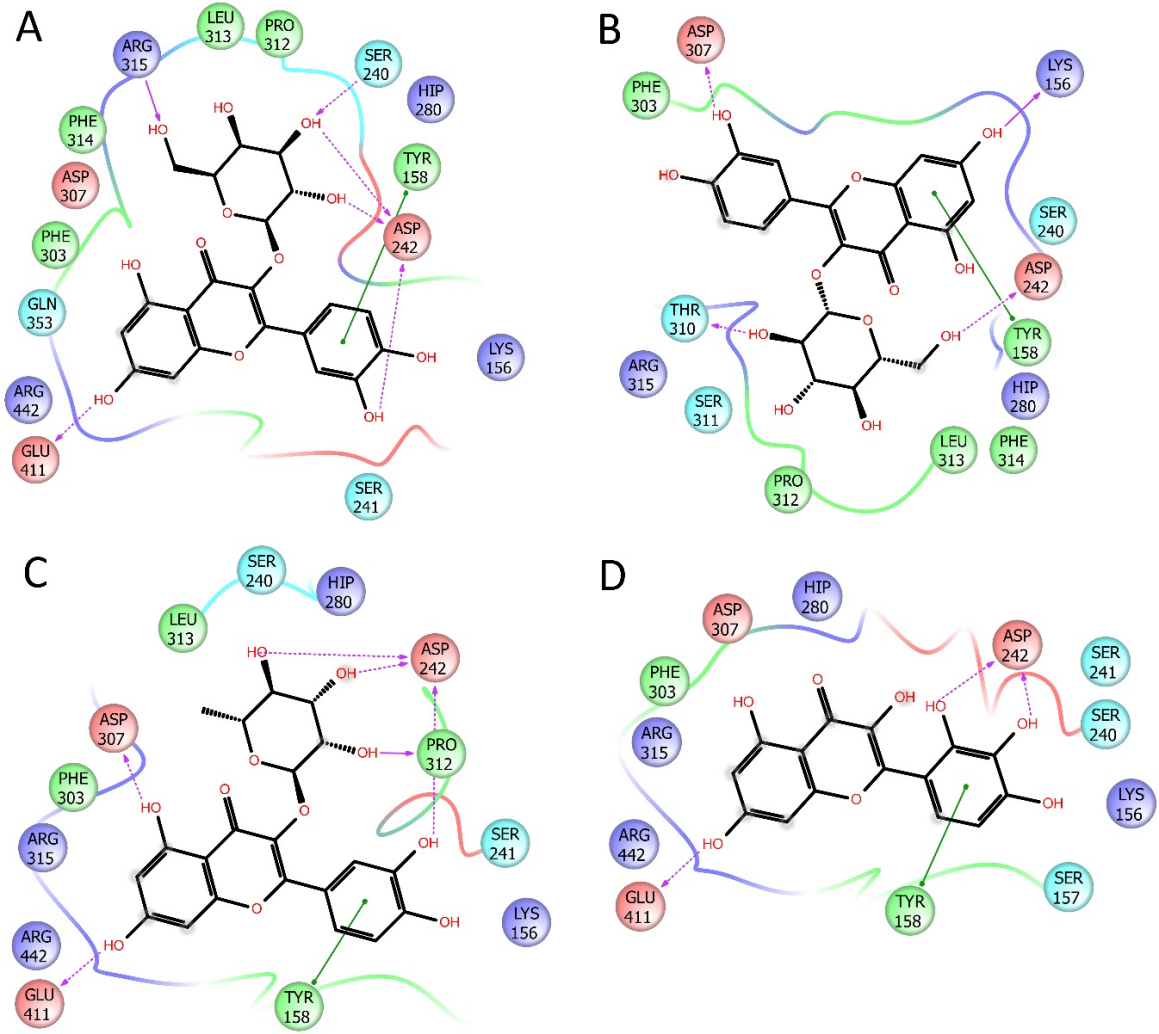
2D representation of the best pose of (A) isoquercetin, (B) isoquercitrin, (C) quercitrin, (D) myricetin docked in the catalitic pocket of α-glucosidase.

The best docking scores for all the selected substances docked into the enzymatic pocket of our enzyme pool are reported in Table 4. We focused the *in silico* experiments only on the most involved enzymes, namely α-glucosidase and tyrosinase. The analysis of the docking scores revealed that the best fitting on tyrosinase was given by the selected flavonols, and partially by the caffeoylquinic acids. We have found caffeoylquinic acid derivatives to give poor interactions with this enzyme by the leaving open to the hypothesis that isoquercitrin, quercetin, isoquercetin, and myricetin-3-*O*-glucoside may be responsible for the excellent inhibition activity found for the extract (Fig 2). Moreover, also the docking study on α-glucosidase, have shown the limited interaction of caffeoylquinic acids to the enzymatic pocket and high docking scores for quercetin, isoquercitrin, quercetin, and myricetin-3-*O*-glucoside (Fig 3). This observation is in agreement with the literature data which reports a good antidiabetic activity of several herbals extracts containing high amount of quercetin [51], isoquercitrin [52], myricetin [53, 54] and their glucosides, by the inhibition of α-glucosidase, and the inhibition of tyrosinase by isoquecitrin and isoquercetin [55, 56].

**Table 4:** Docking scores obtained by Glide XP.

	α-Glucosidase	Tyrosinase
Compounds	Docking Score	Docking Score
Myricetin	-6.11	-6.898
Quercetin	-5.525	-6.959
3-*O*-caffeoylquinic a.	-7.147	-6.091
4-*O*-caffeoylquinic a.	-6.396	-6.698
5-*O*-caffeoylquinic a.	-8.034	-6.060
Myricetin-*O*-hexoside	-7.391	-9.221
Quercetin-*O*-hexoside	-8.221	-7.681
Quercetin	-5.384	-7.198
Isoquercitrin	-7.869	-8.290

### Evaluation of anti-cancer efficacy

*Hypericum* species are one of the most intensely studied groups in the research area of drug design and discovery of natural products for their effective pharmacological contents [57]. Many different *Hypericum* species’ anti-neoplastic effects have been reported in various cancer types, and specific phytochemical libraries were generated for new drug formulations [58–63]. In this study, anti-neoplastic effects of *H*. *salsugineum* were evaluated for the first time.

Breast cancer, which is a heterogeneous and complex disease, is the most common malignancy among women worldwide [64, 65]. Due to variable tissue and receptor activities, patients cannot benefit from the same treatment protocol. Although surgery, chemotherapy, and radiotherapy are the primary treatment modalities in breast cancer, hormone and targeted treatment options are being added according to the progression and type of the disease [66–71]. Despite all these advanced treatment options, efforts are underway to reduce the side effects of the drugs and to develop more effective agents. We investigated the effects of *H*. *salsugineum* on the cellular phenotype of cell lines for the two major molecular subtypes of breast cancer, luminal and basal. MCF-7 and MDA-MB-231, are well known for their clinical, pathological, and immunological characteristics, and are widely used model cell lines in breast cancer research [72]. MCF-7 is a luminal-type breast cancer cell line expressing estrogen receptor and MDA-MB-231 is basal-type cell line and has no hormone receptors such as estrogen receptor, progesterone receptor, and human epidermal growth factor receptor-2. MDA-MB-231 was also reported to be in the Claudin-low class during recent classification studies [73–75].

**Fig 4:**
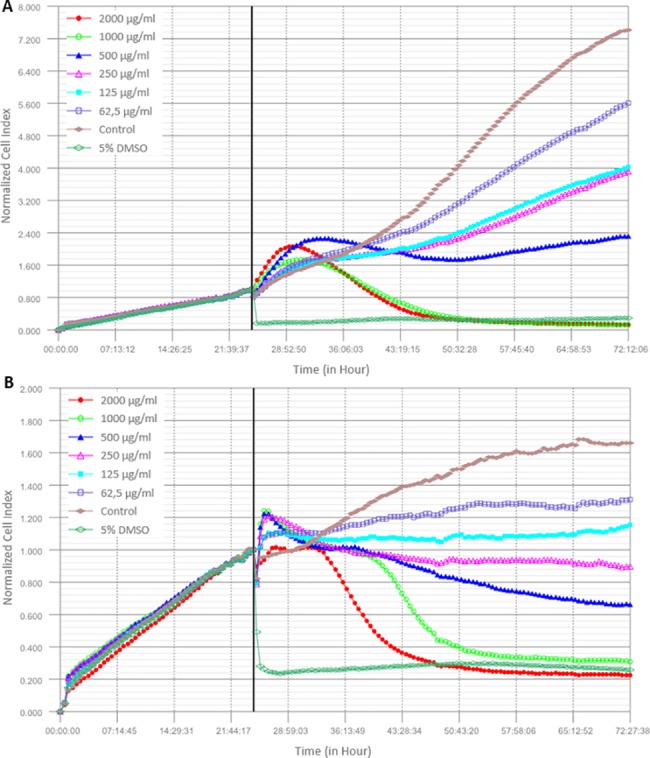
Anti-proliferative and cytotoxic effects of *H*. *salsugineum* methanolic extract on breast cancer cell lines via iCELLigence real time cell analysis system. (A) MCF-7 and (B) MDA-MB-231 cells were treated with varying concentrations (62,5 to 2000 μg/ml) of *H*. *salsugineum* methanolic extract. Charts were represented impedance measurements for 72h in real time and without any additional labelling. IC50 values are the means ± standard deviation of three independent experiments.

We initially investigated the time and dose-dependent effects of *H*. *salsugineum* methanolic extract on the cells by using a real-time, label-free iCELLigence cell analysis system. Platform measures the impedance via gold microelectrodes placed at the bottom of the system-specific E plate L8 and monitors the values in real time, resulting in quantitative high-throughput cellular data [76, 77]. In our study, cells were treated between the range of 62.5 and 2000 μg/ml of *H*. *salsugineum* extract and the effects were recorded in a total of 72 h. According to the obtained cell index values, cells were not viable following 2000 and 1000 μg/ml of *H*. *salsugineum* treatment, whereas an inhibition profile was observed between the range of 500 to 62,5 μg/ml treatments compared to the control (Fig 4). The IC50 values were calculated as 365.3±19.2 and 368±4.2 μg/ml for MCF-7 and MDA-MB-231, respectively. These results demonstrated that *H*. *salsugineum* displayed anti-proliferative effects on cells that were dynamically screened in a serial dose range and obtained IC50 values indicated that it was not cytotoxic to cells.

**Fig 5:**
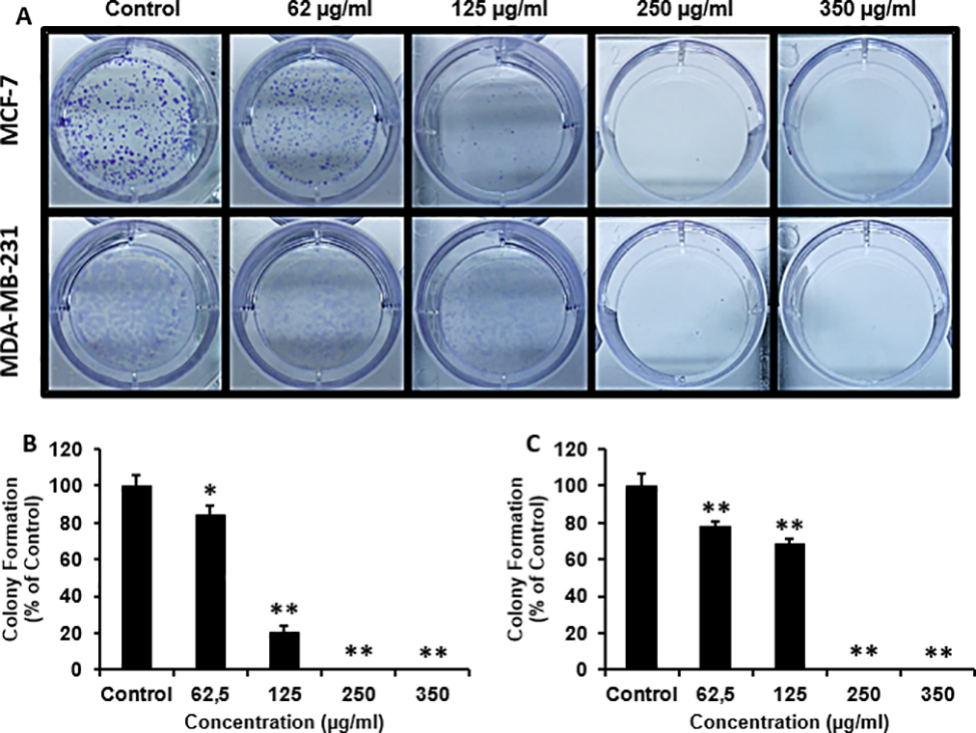
Colony formation ability of breast cancer cell lines by *H*. *salsugineum* methanolic extract treatment. (A) Representative images were showing colony formations for breast cancer cells which were treated with increasing concentrations (62,5 to 350 μg/ml) of *H*. *salsugineum* methanolic extract for 14 days. Histograms show the mean number of colonies in (B) MCF-7 and (C) MDA-MB-231. Values are the means ± standard deviation of three independent experiments. P<0.05 is considered as statistically significant. *P<0.05, **P<0.01 compared to control.

To observe the long-term effects of *H*. *salsugineum* on breast cancer cell lines, colony formation assay was performed. Based on the proliferation data, the cells were treated with doses close to and below the IC50 value of *H*. *salsugineum* extract for 14 days to determine the effect on the colony forming ability. Colony formation in both cell lines was significantly reduced with 62.5 and 125 μg/ml of *H*. *salsugineum* treatment and was completely inhibited with 250 and 350 μg/ml of the extract. *H*. *salsugineum* was more effective on MCF-7 cells than MDA-MB-231 cells for inhibition of colony formation (Fig 5) which may be explained by the more invasive and metastatic characteristics of MDA-MB-231 cell line. However, at 350 μg /ml, which is close to the determined IC50 values, *H*. *salsugineum* displayed evident stochastic effects in both cell lines.

**Fig 6:**
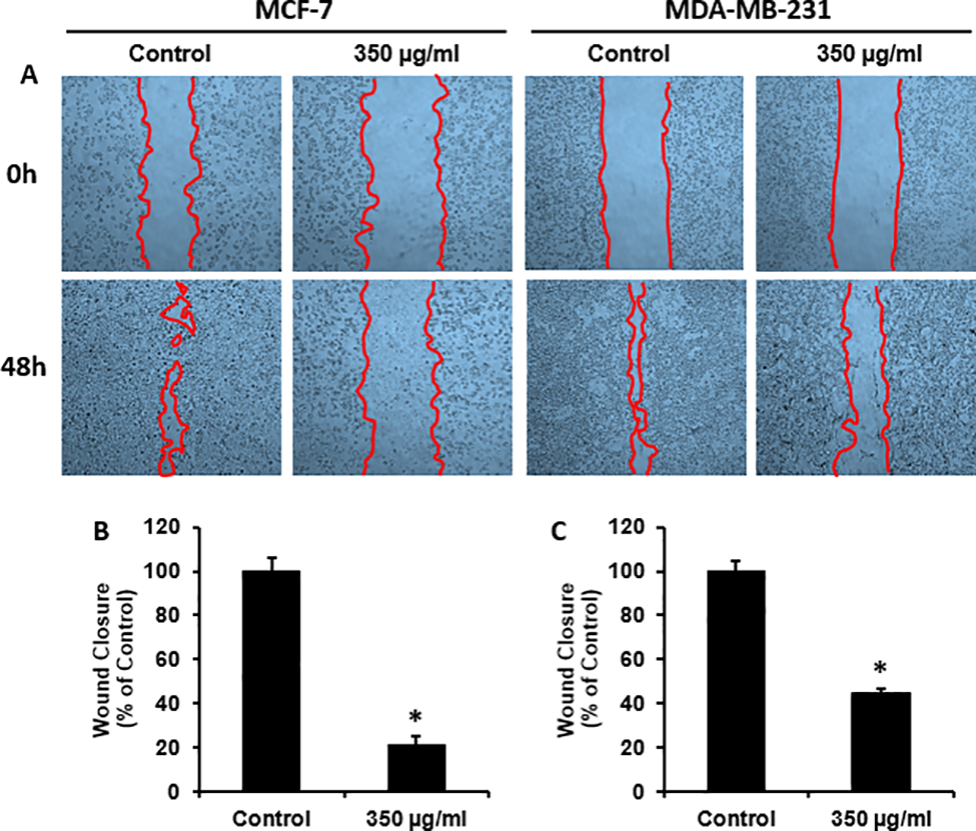
Inhibition of cell migration in breast cancer cell lines by wound healing assay after *H*. *salsugineum* methanolic extract treatment. Cells were scratched and treated with 350 μg/ml *H*. *salsugineum* methanolic extract for 48h. (A) Representative images were indicating wounded areas before and after treating of the cells. Closure rates were analyzed with ImageJ software. Bar graphs shows the mean of closure rates in (B) MCF-7 and (C) MDA-MB-231. The mean values and the ± standard deviation were obtained from three independent experiments. P<0.05 is considered as statistically significant. *P<0.01 compared to control.

For observing the effects of *H*. *salsugineum* on the migration capabilities of MCF-7 and MDA-MB-231 cells, *in vitro* wound healing scratch assay was performed. Fig 6(A) shows the formed wounds at 0 h and the healing progression at 48 h. Cell migration was significantly reduced by 350 μg/ml *H*. *salsugineum* treatment in both cell lines, 21.06±3,9% in MCF-7 and 44.6±2,2% in MDA-MB-231, compared to control (Fig 6B and 6C). Similar to the results of colony formation assay, *H*. *salsugineum* was less effective in MDA-MB-231 cells in reducing the ability to migrate.

The results of our three consecutive assays -proliferation, colony formation, and wound healing- clearly confirmed that *H*. *salsugineum* significantly inhibited cellular growth in MCF-7 and MDA-MB-231 breast cancer cell lines. We believe that the inhibition effects of *H*. *salsugineum* on breast cancer cell lines are due to the compounds that we have identified (Table 2). *H*. *salsugineum* contains chlorogenic acid derivatives as major phenolic compounds, and myricetin and quercetin derivatives as major flavonols. All these phenolic compounds’ chemopreventive properties and mechanisms of action are well-described in the literature [78]. Focusing on recent breast cancer research, Jiao et al. reported that, myricetin suppressed the cell viability of MCF-7 cells mainly through p21-activated kinase 1 through downstream signaling of the β-catenin pathway [79]. In another study, Ranganathan et al. demonstrated that quercetin induces apoptosis in breast cancer cells through suppression of Twist via p38MAPK pathway [80]. Steiner, Davis [81] reported the dose-dependent positive effects of quercetin in transgenic mouse model of human breast cancer and identified the specific gene expression signature related to quercetin treatment. Chlorogenic acid, which is found in the human diet as one of the major active ingredients of many foods, was shown to suppress the proliferative and invasive phenotype of breast cancer cells [82–84]. The results of this study demonstrate that *H*. *salsugineum* methanolic extract has anti-cancer effects on the variable type of breast cancer cell lines with different comprehensive assays. Our findings and earlier research indicate that the inhibitory effects of *H*. *salsugineum* on breast cancer cells may be associated with these compounds and *H*. *salsugineum* can be used for new drug formulations with its bioactive content.

## Conclusion

In recent years, biologically-active compounds have provided great potential as therapeutic agents to control significant health problems, such as Alzheimer’s disease, type II diabetes, and cancer. Within the framework of this information, we focused on the biological potential and chemical characterization of *Hypericum salsugineum*. The tested extract possessed promising biological abilities with high levels of phenolic acids including 3-*O*-caffeoylquinic, 5-*O*-caffeoylquinic, and 4-*O*-caffeoylquinic acids. It significantly inhibited cellular growth in MCF-7 and MDA-MB-231 breast cancer cell lines. The current findings add to a growing body of literature on the genus *Hypericum* and the promising results here reported can provide a basis to design new phytopharmaceuticals and cosmeceuticals from *H*. *salsugineum*. However, further experimental studies (animal or bioavailability studies, etc.) into *H*. *salsugineum* are strongly recommended.
